# Decreased CD127 Expression on CD4+ T-Cells and Elevated Frequencies of CD4+CD25+CD127− T-Cells in Children with Long-Lasting Type 1 Diabetes

**DOI:** 10.1155/2013/459210

**Published:** 2013-11-21

**Authors:** Marcin Moniuszko, Barbara Glowinska-Olszewska, Malgorzata Rusak, Marta Jeznach, Kamil Grubczak, Danuta Lipinska, Robert Milewski, Anna Justyna Milewska, Milena Dabrowska, Ewa Jablonska, Adam Kretowski, Maria Gorska, Anna Bodzenta-Lukaszyk, Artur Bossowski

**Affiliations:** ^1^Department of Regenerative Medicine and Immune Regulation, Medical University of Bialystok, 15-269 Bialystok, Poland; ^2^Department of Allergology and Internal Medicine, University of Bialystok, 15-276 Bialystok, Poland; ^3^Department of Pediatrics, Endocrinology, Diabetology with Cardiology Division, Medical University of Bialystok, 15-274 Bialystok, Poland; ^4^Department of Hematological Diagnostics, Medical University of Bialystok, 15-274 Bialystok, Poland; ^5^Department of Immunology, Medical University of Bialystok, 15-276 Bialystok, Poland; ^6^Department of Endocrinology, Diabetology and Internal Medicine, Medical University of Bialystok, 15-276 Bialystok, Poland; ^7^Department of Statistics and Medical Informatics, Medical University of Bialystok, 15-295 Bialystok, Poland

## Abstract

Pathobiology of type 1 diabetes (T1D) is predominantly associated with T-cell-related actions. Homeostasis of majority of T-cells is critically dependent on signals mediated by CD127 (interleukin-7 receptor, IL-7R). In contrast, regulatory T-cells express very little CD127 and thereby may be delineated by CD4+CD25+CD127− phenotype. Here we aimed to analyze CD127 expression on CD4+ and CD8+ T-cells and enumerate CD4+CD25+CD127− T-cells in long-lasting T1D. T-cells were analyzed by flow cytometry and immunologic data were correlated with vascular, metabolic, and inflammatory parameters. We demonstrated significantly decreased CD127 levels on CD4+, but not CD8+, T cells in T1D pediatric patients. Interestingly, frequencies of CD4+CD25+CD127− T-cells were significantly enhanced in T1D children and correlated well with frequencies of CD34+CD144+ endothelial progenitor cells and CD4+CD25− T-cells. Levels of CD127 on both CD4+ and CD8+ T-cells in T1D patients were not correlated to each other or HbA_1C_. Interestingly, however, CD127 levels on CD4+ T-cells were significantly correlated to frequencies of CD4+CD25+CD127− T-cells, whereas CD127 levels on CD8+ T-cells were significantly correlated to concentrations of VEGF and triglycerides. Our data indicate that CD127 expression is differentially modulated on CD4+ and CD8+ T-cells in the course of T1D. Moreover, we demonstrated that, in contrast to recent-onset T1D, long-lasting T1D is associated with enhancement of T-cells with regulatory phenotype.

## 1. Introduction

Mechanisms of immune dysregulation underlying type 1 diabetes (T1D) are complex and involve a number of intercellular interactions. Destruction of islet beta cells results mainly from T-cell-mediated actions [1, 2]. Despite widely acknowledged contribution of T-cells to T1D pathobiology, our knowledge on phenotypic alterations of these cells in long-lasting T1D remains elusive. One of the most important phenotypic features of CD4+ and CD8+ T-cells directly associated with their function and fate is expression of CD127 (interleukin-7 receptor, IL-7R). CD127-mediated signaling is a nonredundant mechanism of maintaining T cell survival and proliferation. Appropriate responsiveness to IL-7 is warranted by substantial expression of CD127 and accounts for maintaining steady-state numbers of T-cell pool [3–5]. CD127 is not equally expressed among T-cell subsets, with CD4+ T-cells bearing higher levels of CD127 than CD8+ T-cells [6]. In contrast, regulatory CD4+ FoxP3+ T-cells express very little CD127 on their surface and therefore can be easily delineated with the use of flow cytometry by CD4+CD25+CD127− phenotype [7]. Discoveries of last decade proved that Treg cells play an essential role in controlling autoimmunity [8]. In line with these observations, decreased numbers of regulatory T-cells delineated by CD4+CD25+ phenotype were found in pediatric patients with T1D [9]. Lower percentages of CD4+CD127− (but not CD4+CD25+CD127−) T-cells were found in children with newly diagnosed T1D [10, 11]. Similarly, decreased frequencies of CD4+FoxP3+ cells were found in long-lasting T1D [12]. Interestingly, administration of expanded *ex vivo* autologous T-cells with regulatory phenotype, namely, CD4+CD25+CD127−, resulted in prolonged remission of recently diagnosed T1D [13]. To date, however, data on the role and enumeration of CD4+CD25+CD127− T-cells in long-lasting T1D are much more limited. Similarly, little is known about mutual relationships between regulatory T-cells and metabolic parameters or markers of endothelium/vascular injury.

Recently, much attention has been attributed to another mechanism causing CD127 downregulation, namely, T-cell activation. Downregulation of CD127 by T-cell-activating factors has been also demonstrated in a number of animal and *in vitro* models [14]. Correspondingly, we and many other investigators reported decreased levels of CD127 expression on CD4+ and CD8+ T-cells in AIDS [15, 16]. Downregulation of CD127 on entire CD4+ T-cell pool (not only infected CD4+ T-cells) was demonstrated to reflect the status of chronic immune activation characteristic for lentiviral infection [17]. Decreased CD127 levels in HIV-infected individuals are strongly related to increased rate of disease progression, increased T-cell death resulting in CD4+ T-cell loss, and impairment of protective functional immunity [18, 19]. Similarly, we found significantly decreased CD127 on CD4+ T-cells in patients with noninfectious chronic inflammatory diseases characterized by T-cell activation, namely, perennial allergy and asthma [20]. Similarly, alterations of CD127 expression were reported in rheumatoid arthritis patients [21]. Moreover, experimental blockade of CD127 in arthritis mice resulted in significant clinical improvement [22].

To date, despite the crucial role of T-cells in T1D and indispensable role of CD127 for T-cell function, CD127 expression has never been studied in T1D patients. Precise evaluation of CD127 levels on T-cells could provide not only an insight into biology of CD4+ and CD8+ T-cells in T1D but might have important therapeutic implications also. Recently, two independent experimental studies in nonobese diabetes (NOD) mice provided an elegant evidence that the CD127 blockade induced complete remission and reverted autoimmune diabetes by modulating T-cell function [23, 24]. On the other hand, long-lasting T1D is associated with the high risk of chronic vascular complications and the vascular injury has been recently linked to alterations of immune system [25–27]. Long-lasting T1D is also accompanied by increased prevalence of autoimmune thyroid or celiac disease [28, 29]. Given promising data in recent-onset T1D, it would be of interest to evaluate whether CD127− or Treg-based therapies could also modify the risk of development of T1D-related complications. To make such therapeutic approaches realistic, detailed assessment of their pharmacological target in humans, namely, CD127, would be required. Therefore, in the current study we aimed to evaluate CD127 levels on both CD4+ and CD8+ T-cells in combination with putative Treg cell levels in a representative cohort of pediatric patients with long-lasting T1D, without clinically apparent complications and additional diseases, in a broad context of metabolic, vascular, and inflammatory parameters.

## 2. Material and Methods

### 2.1. Patients

We recruited for the study thirty-three children with diabetes type 1, with history of the disease duration of at least two years (aged 14.3 ± 2.6; range: 10–18 years, mean diabetes duration: 7.0 ± 2.8 years; mean HbA_1C_ level during last 6 months: 8.8 ± 1.5% (72.7 ± 16.4 mmol/mol)). Type 1 diabetes was recognized according to American Diabetes Association criteria. All patients were on insulin treatment, either on multiple insulin injections or continuous subcutaneous insulin infusion. Inclusion criteria for the study group were as follows: insulin requirement: at least 0.5 IU/kg/day, age below 18 and above 9 years and absence of any additional autoimmune disease (e.g., thyroid, celiac disease), as well as absence of chronic complications: albuminuria, retinopathy, or neuropathy. The control group included fifty-two age- and gender-matched children with negative family history of CVD and absence of systemic inflammatory disease based on physical and laboratory examination. Controls were recruited from patients admitted to our hospital due to minor cardiologic problems who were otherwise healthy. The pubertal development was determined by the same pediatrician endocrinologist (AB), according to Tanner classification and participants were categorized into prepubertal (Tanner stage 1) or pubertal (stages 2–5). All studied children were of Caucasian origin. Basic characteristic of the study groups is presented in Table 1. The study was approved by the Ethical Committee of the Medical University of Bialystok. Both parents/legal guardians and children gave their written informed consent.

### 2.2. Laboratory Investigations

Blood sample of 10 mL was taken from the left cubital vein, after an overnight (8–12 h) fast. Lipids were determined by standard enzymatic methods (Hitachi 912, La Roche, Japan). LDL concentration was assessed by the Friedewald equation. HbA_1C_ level was measured using HPLC method. To assess vascular biomarkers serum samples were collected, frozen, and stored at the temperature of −80°C until analyses were performed. The concentrations of VEGF, VE-cadherin and angiopoietin were determined immunoenzymatically using commercially available ELISA kits (Parameter Human Immunoassays, R&D Systems, Inc., Minneapolis, USA, with the use of ELx 800 Automated Microplate Reader, Bio-Tek Instruments, Vermont, USA). hsCRP was determined with use of immunoturbidimetric method (Tina-quant hsCRP (Latex) HS, Roche; Hitachi 912, La Roche, Japan).

### 2.3. Ultrasound Vascular Measurements

Examinations of the brachial and carotid arteries were performed with Hewlett Packard Sonos 4500 apparatus, using a 7.5 MHz linear transducer. The procedure was conducted between 8.00 and 10.00 AM after a fasting period of 8–12 hours. Measurements of intimamedia thickness (IMT) in the common carotid arteries (right and left) and examinations of the right brachial artery reactivity-flow-mediated dilation (FMD) were performed as described previously [30, 31].

### 2.4. Flow Cytometry Analysis

**Figure 1:**
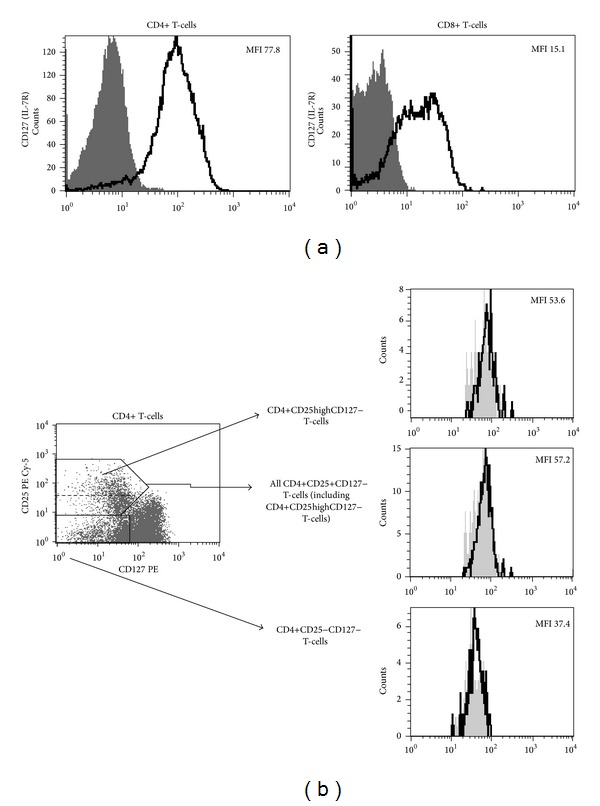
Representative histograms and plots characteristic for studied individuals. (a) Flow cytometric analysis of mean fluorescence intensity of CD127 expression on CD4+ and CD8+ T-cells. Thick black lines represent staining with anti-CD127 monoclonal antibody, grey areas represent fluorescence-minus-one controls. (b) Left panel: dot plot demonstrates staining method used for delineation of CD4+CD25+CD127− T-cells (upper black box) and CD4+CD25-CD127− T-cells (bottom black box). Dashed line within upper black box delineates CD4+CD25highCD127 T-cells within all CD4+CD25+CD127− T-cells. Right panel: histograms representing FoxP3 expression within different T-cell subsets (black lines). Grey areas represent isotype controls. Values are for mean fluorescence intensity (MFI) of FoxP3 expression within different T-cells subsets.

Fresh whole blood samples were incubated for 30 min. at room temperature with 5 *μ*L of the following mAbs from BD PharMingen, Belgium: anti-CD3 FITC, anti-CD4 FITC, anti-CD8 PE, anti-CD25 PE-Cy5 (IL-2R), anti-CD127 AlexaFluor 647, or anti-CD127 PE (IL-7R, Immunotech, France) and appropriate fluorescence-minus-one (FMO) and isotype controls. For analysis of endothelial progenitor cells (EPCs), fresh whole blood EDTA-anticoagulated samples (100 *μ*L) were incubated for 30 minutes at room temperature with the following monoclonal antibodies: 20 *μ*L of FITC anti-human CD 34, 5 *μ*L of PE anti-human CD144 (VE-cadherin), or 5 *μ*L of PE anti-human CD309 (VEGFR-2) (BD PharMingen, Erembodegen, Belgium). The cells were lysed with BD FACS Lysing Solution (BD Immunocytometry Systems), washed twice with phosphate-buffered saline, and fixed with CellFix (BD Immunocytometry Systems). Flow cytometry analysis was performed with the use of a FACSCalibur cytometer (BD Immunocytometry Systems). In order to ensure the reproducibility of the data generated, the settings and calibration of the instrument fluorescence detectors were monitored and optimized according to manufacturer's recommendations, using CaliBRITE beads (BD Immunocytometry Systems). Flow cytometry data were collected in list mode and analyzed using CellQuest software (BD Immunocytometry Systems). Due to monophasic distribution of CD127, data on its expression have been presented as mean fluorescence intensity (MFI) (Figure 1(a)). Analysis of EPCs was performed with the use of flow cytometry based on the surface expression of the following markers: CD34, CD144, and CD309 on the cells localized in the lymphocyte and monocyte gates. Based on initial analysis of FMO controls, circulating progenitor cells were next identified as cells CD34-positive cells and EPCs were next identified as either CD34+VE-cadherin+ (CD34+CD144+) or CD34+VEGFR-2+ (CD34+CD309+) cells. Results regarding EPCs are presented as percentage of total viable mononuclear cells.

### 2.5. Statistical Analysis

Statistical analysis was performed with the use of GraphPrism software (USA). All continuous variables were tested for normal distribution by the Kolmogorov-Smirnov, with Lilliefors correction and Shapiro-Wilk tests. Unpaired Student *t*-test was used for normally distributed variables and Mann-Whitney *U*-test was applied for samples not fitting parameterized distribution to compare the differences between two groups. Correlations between variables of interest were assessed by Spearman's rank coefficient test. Data are expressed as either mean ± SD or median (interquartile range—IQR). The level of statistical significance was set at *P* < 0.05.

## 3. Results

### 3.1. CD127 Expression Is Decreased on CD4+ T-Cells of T1D Children

**Figure 2:**
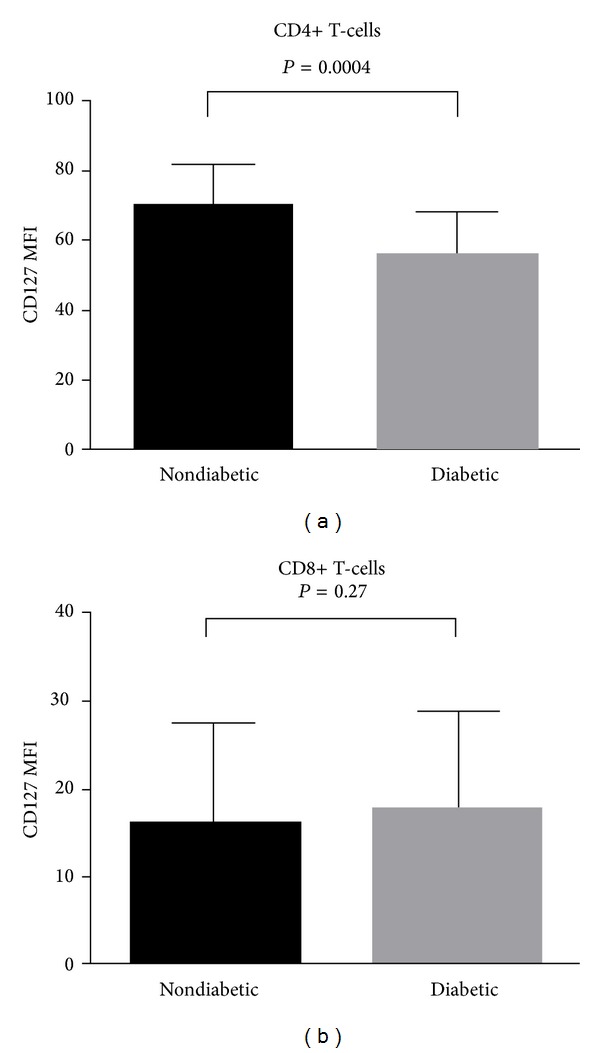
Comparative analysis of CD127 expression on CD4+ and CD8+ T-cells in healthy controls and T1D pediatric patients. Bars represent medians while whiskers represent interquartile range. MFI: mean fluorescence intensity.

First, we aimed to investigate levels of CD127 expression on the surface of CD4+ and CD8+ T-cells collected from children with long-lasting (range 2–14 years) T1D in comparison to nondiabetic children. Notably, CD4+ T-cells of T1D patients expressed significantly less CD127 than healthy children (56.15 [45.03–68.48] versus 70.85 [59.10–81.80], respectively, *P* = 0.0004) (Figure 2, Table 2). In contrast, this pattern was not observed for CD127 levels on CD8+ T-cells in T1D children. In fact, CD127 expression on CD8+ T-cells tended to reach higher values in T1D patients than in healthy subjects (17.92 [14.94–24.93] versus 15.88 [14.89–19.08], resp., *P* = 0.27). Interestingly, and, in contrast to our previous observations in lentiviral infection, CD127 levels on CD4+ and CD8+ T-cells were not correlated to each other (*R* = 0.17, *P* = 0.44).

### 3.2. Relationships between CD127 Expression and Metabolic, Inflammatory, and Vascular Parameters

Having found contrasting profiles of CD127 expression on CD4+ and CD8+ T-cells, we next wished to investigate whether such phenotypic pattern of T-cells in T1D can be related to other markers and parameters associated with T1D. Thus we performed a comprehensive analysis of correlations between CD127 levels on CD4+ and CD8+ T-cells and patients' age, BMI, age of disease's onset, duration of disease, mean HbA_1C_ levels (from last six months), hsCRP levels, white blood counts, systolic and diastolic blood pressure, and concentrations of VEGF, VE-cadherin, angiopoietin, cholesterol, LDL, HDL, and TG. In addition, following our recent observation of enhanced levels of endothelial progenitor cells (EPCs) in T1D children, we correlated CD127 levels with frequencies of CD34+CD144+ and CD34+CD309+ cells [30]. Finally, we correlated CD127 levels with frequencies of putative effector T-cells with CD4+CD25− phenotype. Detailed analysis of all correlations is presented in Table 3 (first and middle panels). Again, we observed differential pattern of relationships for CD4+ and CD8+ T-cells. Neither of abovementioned metabolic, inflammatory, and vascular/endothelial parameters was correlated to CD127 levels on CD4+ T-cells. In some contrast, CD127 levels on CD8+ T-cells were positively correlated to concentrations of VEGF and TG (*P* = 0.04 for both correlations).

### 3.3. Assessment of Frequencies of CD4+CD25+CD127− T-Cells in T1D

**Figure 3:**
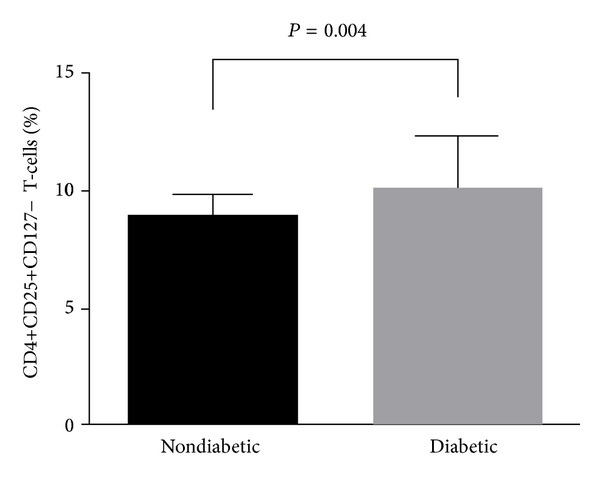
Comparative analysis of frequencies of CD4+CD25+CD127− T-cells in healthy children and T1D children. Bars represent medians while whiskers represent interquartile range.

One of the most precise phenotypic assessment of T-cells with regulatory phenotype can be performed with the use of combination of monoclonal antibodies directed against CD4, CD25 (IL-2 receptor alpha), and CD127. Such method allows for delineation of putative T regulatory cells, namely, CD4+CD25+CD127− T-cells (Figure 1(b)). CD4+CD25+CD127 T-cells express high levels of FoxP3 and exert highly immunosuppressive effects directed against effector T-cells [7, 8]. Most recently, clinical administration of CD4+CD25+CD127− T-cells has been shown to prolong remission of freshly diagnosed T1D [13] Here, we performed cross-sectional analysis of CD4+CD25+CD127− T-cells in long-lasting T1D. Surprisingly, frequencies of CD4+CD25+CD127− T-cells were significantly higher in T1D patients when compared with healthy controls (10.02 [8.23–12.13] versus 8.89 [7.20–9.87], resp., *P* = 0.004) (Figure 3). Similarly, we observed higher frequencies of CD4+CD25highCD127− T-cells in T1D patients (data not shown). Notably, frequencies of CD4+CD25+CD127− T-cells were negatively correlated with CD127 levels on CD4+, but not CD8+, T-cells.

### 3.4. Relationships between T-Cells with Regulatory Phenotype and Metabolic, Inflammatory and Vascular/Endothelial Parameters of T1D

Despite broad interest in role of Treg cells in pathogenesis of T1D, their role has not been studied in detail in the context of other markers indicating progression and/or complications of long-lasting disease. Here we performed an extensive analysis of relationships between CD4+CD25+CD127− T-cells and a number of metabolic and vascular/endothelial parameters (Table 3, bottom panel). Interestingly, we demonstrated that frequencies of CD4+CD25+CD127− T-cells were positively correlated to CD127 expression on CD4+ T-cells but negatively correlated to frequencies of CD4+CD25− T-cells (*P* = 0.01 and *P* < 0.001, resp.). Similar significant correlations were found also for CD4+CD25highCD127− T-cells (data not shown). In addition we found that CD4+CD25+CD127− T-cells were significantly related to frequencies of EPCs delineated by CD34+CD144+ phenotype (*P* = 0.04).

## 4. Discussion

In this study, for the first time we assessed levels of CD127 expression on circulating CD4+ and CD8+ T-cells in T1D in relation to comprehensively analyzed metabolic, inflammatory, and vascular parameters of T1D. Our data indicate for differential regulation of phenotype of CD4+ and CD8+ T-cells in long-lasting T1D. This complements our current knowledge stating that both subsets play different roles in pathogenesis of T1D [32]. Our previous observations in lentiviral infection strongly indicated that levels of CD127 expression on CD4+ and CD8+ T-cells are strongly correlated to each other [15]. Lack of such correlation observed in T1D indicates differential mechanisms affecting CD4+ and CD8+ T-cells. Our demonstration of higher CD127 levels on CD8+ than on CD4+ T-cells is in concert with previous studies indicating that CCR7 levels in recently diagnosed T1D children are increased on CD8+, but not CD4+, T-cells [33]. Notably, CCR7 is mainly expressed on naïve and central memory T-cells, both subsets being also enriched in CD127. In contrast, effector T-cells express little CD127 and CCR7. Thus, based on our data, we could hypothesize that effector CD8+ T-cells might be relatively deficient in T1D pediatric patients. Further studies are warranted to explore whether such decrease in CD8+ T-cell effector function could contribute to immunopathology of T1D.

Having analyzed our immunologic data we initially hypothesized that contrasting profiles of CD127 expression on CD4+ and CD8+ T-cells could be the consequence of differential interactions with altered metabolic and/or inflammatory status characteristic for T1D. However, lack of correlations between CD127 levels on either CD4+ or CD8+ T-cells and HbA_1C_, BMI, hsCRP, and white blood cell counts does not seem to confirm such hypothesis. Out of numerous analyzed parameters, CD127 levels on CD4+ T-cells were correlated only to frequencies of T-cells with regulatory CD4+CD25+CD127− phenotype. Thus, decreased CD127 expression on CD4+ T-cells in T1D children could be at least in part explained by enrichment of CD127−negative putative regulatory CD4+ T-cells. The latter finding is actually quite surprising given the current knowledge on the role of regulatory T-cells in T1D and, more generally, in autoimmunity. Numerous reports indicate that autoimmune disorders, including T1D, are characterized by decrease in regulatory T-cell numbers [9, 10, 34, 35]. With regard to T1D, however, majority of such data derive from recently diagnosed disease. These studies are however contrasted by a number of reports describing the opposite pattern of Treg cells in the course of autoimmune disorders [36, 37].

Our study is one of very few reports addressing the issue of CD4+CD25+CD127− T-cells in long-lasting T1D and, notably, in the context of metabolic and early vascular consequences of T1D in young population without clinically apparent cardiovascular disease. Recently, Ryba-Stanislawowska et al. reported altered balance between Treg cells and Th17 cells and its association with development of retinopathy in patients with long-standing T1D [38]. Similarly, relationships between dysregulated Treg/Th17 balance and metabolic complications were found in diabetes type 2 patients [39]. Here we found positive relationship between frequencies of CD4+CD25+CD127− T-cells and frequencies of endothelial progenitor cells delineated by CD34+CD144+ phenotype. EPCs can be delineated by varying markers including CD133, CD144, or CD309. We recently demonstrated that CD34+CD144+ subset is significantly enriched in T1D patients. To date, correlations between endothelial progenitor cells and putative Treg cells have never been studied in T1D. One historical study performed in healthy subjects demonstrated positive relationship between numbers of CD4+CD25high T-cells and CD34+CD309+ progenitor cells [40]. Given these and our data, it is possible that increased levels of T-cells with regulatory phenotype could contribute to promoting EPC recruitment. On the other hand, our demonstration of enhanced levels of CD4+CD25+CD127− T-cells in long-lasting T1D could represent compensative mechanisms developed by immune system in reaction to prolonged autoimmune pathology. This hypothesis stems from strong negative correlation between CD4+CD25+CD127− T-cells and CD4+CD25− T-cells observed in our study. Importantly, majority of CD4+ T-cells responsible for pancreatic beta-cell destruction in T1D have been shown previously to present with CD4+CD25− phenotype [41]. In line with our observations, enhanced levels of certain subsets of regulatory T-cells were observed in active SLE [42]. Similarly, enhanced numbers of fully suppressive Treg cells were identified in synovial tissue of patients with chronic rheumatoid arthritis [43]. Finally, some groups reported no statistical differences in numbers of Treg cells in autoimmune disorders such as SLE or T1D [44, 45]. In general, our finding of elevated CD4+CD25+CD127− T-cells in long-lasting T1D provides another evidence for lack of uniform quantitative pattern of Treg cells in the course of autoimmune diseases. From therapeutical point of view, it would be of interest however to examine whether children with long-lasting T1D (that are enriched in CD4+CD25+CD127− T-cells) could benefit from transplantation of these cells at the level similar to children with newly diagnosed T1D [13]. Results of our study could help building a platform for future immune-based interventions aiming to improve endothelial function and decrease vascular complications in the course of long-lasting T1D [46, 47].

As CD127 is downregulated following T-cell activation, another explanation of decreased CD127 expression on CD4+ T-cells in long-lasting T1D could be selective activation/stimulation of this cell subset. Interestingly, pattern of decreased CD127 expression observed here in T1D pediatric patients resembles that observed in our previous studies on lentiviral infection [15]. In general, decreased CD127 expression is being considered a marker of chronic immune activation. To date, however, reports on CD4+ T-cell activation in T1D (and more specifically in long-lasting T1D) are scarce and bring conflicting results. Yang et al. demonstrated decreased thresholds of activation in CD4+ T-cells derived from T1D patients [48]. In some contrast, Aarnisalo et al. reported reduced activation of CD4+ T-cells in T1D pediatric patients carrying the PTPN22/Lyp 620Trp variant [49]. On the other hand, report of Baker et al. demonstrated expansion of small subpopulation of CD3+CD30+DR+ cells (without specifying differences between CD4+ and CD8+ T-cells) in newly diagnosed but not long-lasting adult T1D patients [50]. Despite these rare reports, our current knowledge on phenotypic alterations of CD4+ and CD8+ T-cells in long-lasting pediatric T1D is very limited. Thus, our data on differential expression of one of crucial T-cell-associated molecules (CD127) warrant further studies on more detailed phenotypic characterization of T-cells in long-lasting T1D. Similarly, additional studies are needed to better quantify whether decreased expression of receptor for IL-7 on CD4+ T-cells results in a shorter life-span of these cells in T1D patients.

One of the most intriguing observations of our study is finding significant correlations between CD127 expression on CD8+ T-cells and mediator released in response to endothelial/vascular damage, namely, VEGF. To date, the specific mechanisms underlying development of vascular injury in T1D have not been fully elucidated. One of hypotheses states that dysregulated autoimmune responses could not only lead to pancreatic beta cell destruction but also to vascular endothelial dysfunction [41]. However, current data on potential associations between alterations of immune system and endothelium are very limited and in majority come from other than T1D disorders. Study on lung cancer cells provided evidence that IL-7/IL-7R is well correlated with VEGF and induce its upregulation [51]. On the other hand, Yadav et al. demonstrated significant relationship between intimamedia thickness and certain subsets of CD4+ T-cells (e.g. CD4+CD28null) in chronic kidney disease patients [52]. Less is known about such immune-endothelial interactions in T1D patients. Our demonstration of correlations between VEGF and phenotypic features of CD8+ T-cells, but not CD4+ T-cells and putative Treg cells, could suggest that CD8+ T-cells could at least in part be implicated in regulation of vascular injury/repair in the course of long-lasting T1D.

Our comprehensive analysis of CD127 expression on different T-cell subsets not only increases our knowledge on alterations of T-cell-mediated immunity in T1D but also provides potentially useful information on therapeutic targets for novel T-cell-directed therapies. Given very promising data on the use of CD127 blockade in experimental autoimmune diabetes, we could hypothesize that CD4+ and CD8+ T-cells could present with varying susceptibility to CD127 blockade due to differential levels of CD127. Further studies will be warranted to explore whether CD8+ T-cells can be a potential better target than CD4+ T-cells for CD127−related immune interventions in T1D.

## 5. Conclusions

Altogether, we presented here evidences of novel phenotypic alterations of CD4+ and CD8+ T-cells in the context of metabolic and vascular/endothelial parameters in long-lasting T1D. In addition, we demonstrated that long-lasting T1D, in contrast to recently diagnosed T1D, is characterized by distinct quantitative pattern of T-cells with regulatory phenotype. 

## Figures and Tables

**Table 1:** General characteristics of study groups. Data are presented as mean ± standard deviation or median (interquartile range) for normally and nonnormally distributed data, respectively.

	Study group	Control group	*P* value
Number of patients	33	52	
Gender (boys/girls), *n* (%)	16 (48)/17 (52)	28 (54)/24 (46)	
Age (yrs)	14.3 ± 2.6	14.6 ± 2.7	0.55
Height (m)	1.6 ± 0.1	1.6 ± 0.1	0.88
Body mass (kg)	59.5 ± 13.7	54.9 ± 13.4	0.12
Body mass index (kg/m^2^)	22.1 ± 3.3	20.2 ± 2.8	<0.001
SDS-BMI	1.0 ± 1.0	0.3 ± 0.9	0.01
Age of onset (yrs)	7.3 ± 3.2		
Disease duration (yrs)	7.0 ± 2.8		
HbA_1c_ level	8.8 ± 1.8% (72.7 ± 19.7 mmol/mol)		
HbA_1c_ level (mean from last 6 months)	8.8 ± 1.5% (72.7 ± 16.4 mmol/mol)		
Systolic blood pressure (mmHg)	115 ± 9	115 ± 11	0.86
Diastolic blood pressure (mmHg)	70 ± 6	68 ± 9	0.45
Total cholesterol (mmol/L)	4.6 ± 0.7	4.1 ± 0.6	0.01
LDL-cholesterol (mmol/L)	2.4 ± 0.6	2.3 ± 0.5	0.3
HDL-cholesterol (mmol/L)	1.6 ± 0.3	1.3 ± 0.3	<0.001
Triglycerides (mmol/L)	0.7 (0.6–1)	0.8 (0.5–1.1)	0.8
IMT (mm)	0.49 ± 0.05	0.43 ± 0.06	<0.001
FMD (%)	7.1 ± 5.1	10.8 ± 4	0.007

**Table 2:** Analysis of mutual relationships among CD127 expression on CD4+ and CD8+ T-cells and percentage of CD4+CD25+CD127− T-cells in T1D children.

Correlations	*P* value, *R *
CD127 MFI on CD4+ T-cells versus CD127 MFI on CD8+ T-cells	*P* = 0.44, *R* = 0.17
**CD127 MFI on CD4**+** T-cells versus** **% of CD4**+**CD25**+**CD127**−** T-cells**	***P* = 0.01, *R* = −0.42**
CD127 MFI on CD8+ T-cells versus % of CD4+CD25+CD127− T-cells	*P* = 0.59, *R* = −0.12

Statistically significant correlations are indicated in bold font.

**Table 3:** Comparison of correlations among CD127 expression on CD4+ (first part of the table) and CD8+ (middle part of the table) T-cells and frequencies of CD4+CD25+CD127− T-cells (last part of the table) and metabolic, inflammatory, and vascular/endothelial parameters in children with long-lasting T1D.

Correlations of CD127 MFI on CD4+ T-cells	*P* value, *R *
CD127 MFI versus age	*P* = 0.48, *R* = 0.13
CD127 MFI versus duration of disease	*P* = 0.34, *R* = 0.17
CD127 MFI versus current HbA_1c_	*P* = 0.47, *R* = 0.13
CD127 MFI versus mean HbA_1C_ (from last 6 months)	*P* = 0.20, *R* = 0.23
CD127 MFI versus BMI	*P* = 0.15, *R* = 0.26
CD127 MFI versus mean SBP	*P* = 0.89, *R* = −0.02
CD127 MFI versus mean DBP	*P* = 0.19, *R* = 0.23
CD127 MFI versus white blood cells	*P* = 0.30, *R* = −0.21
CD127 MFI versus hsCRP	*P* = 0.18, *R* = −0.28
CD127 MFI versus VE-cadherin	*P* = 0.67, *R* = −0.09
CD127 MFI versus angiopoietin	*P* = 0.07, *R* = 0.37
CD127 MFI versus VEGF	*P* = 0.32, *R* = −0.21
CD127 MFI versus IMT	*P* = 0.93, *R* = 0.02
CD127 MFI versus FMD	*P* = 0.88, *R* = −0.03
CD127 MFI versus cholesterol	*P* = 0.07, *R* = 0.32
CD127 MFI versus LDL	*P* = 0.10, *R* = 0.30
CD127 MFI versus HDL	*P* = 0.48, *R* = 0.13
CD127 MFI versus TG	*P* = 0.27, *R* = 0.20
CD127 MFI versus % of CD34+CD309+ cells	*P* = 0.37, *R* = −0.19
CD127 MFI versus % of CD34+CD144+ cells	*P* = 0.88, *R* = 0.03
CD127 MFI versus % of CD4+CD25− T-cells	*P* = 0.94, *R* = −0.01

Correlations of CD127 MFI on CD8+ T-cells	*P* value, *R *

CD127 MFI versus age	*P* = 0.16, *R* = 0.30
CD127 MFI versus duration of disease	*P* = 0.48, *R* = −0.15
CD127 MFI versus current HbA_1c_	*P* = 0.96, *R* = 0.01
CD127 MFI versus mean HbA_1C_ (from last 6 months)	*P* = 0.70, *R* = −0.09
CD127 MFI versus BMI	*P* = 0.43, *R* = 0.17
CD127 MFI versus mean SBP	*P* = 0.88, *R* = 0.03
CD127 MFI versus mean DBP	*P* = 0.91, *R* = 0.03
CD127 MFI versus white blood cells	*P* = 0.69, *R* = 0.09
CD127 MFI versus hsCRP	*P* = 0.94, *R* = −0.02
CD127 MFI versus VE-cadherin	*P* = 0.12, *R* = −0.36
CD127 MFI versus angiopoietin	*P* = 0.05, *R* = 0.44
**CD127 MFI versus VEGF**	***P* = 0.04, *R* = 0.46**
CD127 MFI versus IMT	*P* = 0.44, *R* = −0.19
CD127 MFI versus FMD	*P* = 0.37, *R* = −0.22
CD127 MFI versus cholesterol	*P* = 0.47, *R* = 0.16
CD127 MFI versus LDL	*P* = 0.47, *R* = 0.16
CD127 MFI versus HDL	*P* = 0.20, *R* = −0.28
**CD127 MFI versus TG**	***P* < 0.05, *R* = 0.43**
CD127 MFI versus % of CD34+CD309+ cells	*P* = 0.60, *R* = 0.13
CD127 MFI versus % of CD34+CD144+ T-cells	*P* = 0.89, *R* = −0.03
CD127 MFI versus % of CD4+CD25− T-cells	*P* = 0.75, *R* = 0.07

Correlations of % of CD4+CD25+CD127− T-cells	*P* value, *R *

% of CD4+CD25+CD127− T-cells versus age	*P* = 0.32, *R* = 0.18
% of CD4+CD25+CD127− T-cells versus duration of disease	*P* = 0.11, *R* = 0.28
% of CD4+CD25+CD127− T-cells versus current HbA_1c_	*P* = 0.23, *R* = 0.22
% of CD4+CD25+CD127− T-cells versus mean HbA_1C_ (from last 6 months)	*P* = 0.30, *R* = 0.19
% of CD4+CD25+CD127− T-cells versus BMI	*P* = 0.62, *R* = 0.09
**% of CD4**+**CD25**+**CD127**−** T-cells versus mean SBP**	***P* = 0.04, *R* = 0.35**
% of CD4+CD25+CD127− T-cells versus mean DBP	*P* = 0.52, *R* = 0.12
% of CD4+CD25+CD127− T-cells versus white blood cells	*P* = 0.90, *R* = −0.03
% of CD4+CD25+CD127− T-cells versus hsCRP	*P* = 0.99, *R* = 0.00
% of CD4+CD25+CD127− T-cells versus VE-cadherin	*P* = 0.78, *R* = −0.06
% of CD4+CD25+CD127− T-cells versus angiopoietin	*P* = 0.47, *R* = −0.15
% of CD4+CD25+CD127− T-cells versus VEGF	*P* = 0.84, *R* = 0.04
% of CD4+CD25+CD127− T-cells versus IMT	*P* = 0.61, *R* = 0.11
% of CD4+CD25+CD127− T-cells versus FMD	*P* = 0.90, *R* = 0.03
% of CD4+CD25+CD127− T-cells versus cholesterol	*P* = 0.68, *R* = 0.07
% of CD4+CD25+CD127− T-cells versus LDL	*P* = 0.60, *R* = 0.10
% of CD4+CD25+CD127− T-cells versus HDL	*P* = 0.36, *R* = −0.17
% of CD4+CD25+CD127− T-cells versus TG	*P* = 0.57, *R* = −0.10
% of CD4+CD25+CD127− T-cells versus % of CD34+CD309+ cells	*P* = 0.39, *R* = 0.18
**% of CD4**+**CD25**+**CD127**−** T-cells versus % of CD34**+**CD144**+** cells**	***P* = 0.04, *R* = 0.43**
**% of CD4**+**CD25**+**CD127**−** T-cells versus % of CD4**+**CD25**−** T-cells**	***P* < 0.001, *R* = −0.81**

SBP: systolic blood pressure; DBP: diastolic blood pressure; VEGF: vascular endothelial growth factor; IMT: intimamedia thickness; FMD: flow-mediated dilation; LDL: low density lipoproteins; HDL: high density lipoproteins; TG: triglycerides. Statistically significant correlations are indicated in bold font.

## Abbreviations

CCR7: C-C chemokine receptor type 7
CVD: Cardiovascular disease
EPC: Endothelial progenitor cell
FMD: Flow-mediated dilation
FMO: Fluorescence minus one
hsCRP: High-sensitivity C-reactive protein
IMT: Intimamedia thickness
mAb: Monoclonal antibody
PE: R-phycoerythrin
SLE: Systemic lupus erythematosus
T1D: Type 1 diabetes
TG: Triglyceride
VEGF: Vascular endothelial growth factor
WBC: White blood cell.
